# Dramatically Enhancing the Sensitivity of Immunoassay for Ochratoxin A Detection by Cascade-Amplifying Enzyme Loading

**DOI:** 10.3390/toxins13110781

**Published:** 2021-11-05

**Authors:** Zhuolin Song, Lin Feng, Yuankui Leng, Mingzhu Huang, Hao Fang, Weipeng Tong, Xuelan Chen, Yonghua Xiong

**Affiliations:** 1 College of Life Science, Jiangxi Normal University, Nanchang 330022, China; zhuolinsong0508@163.com (Z.S.); linfeng2021102021@163.com (L.F.); huangmingzhu12@163.com (M.H.); 2 State Key Laboratory of Food Science and Technology, Nanchang University, Nanchang 330047, China; 357900210001@email.nuc.edu.cn (H.F.); 40231337519017@email.ncu.edu.cn (W.T.); xiongyonghua@ncu.edu.cn (Y.X.)

**Keywords:** cascade-amplifying enzyme loading, M13 bacteriophage, polymeric horseradish peroxidases, ochratoxin A

## Abstract

Enzyme-linked immunosorbent assay (ELISA) is widely used in the routine screening of mycotoxin contamination in various agricultural and food products. Herein, a cascade-amplifying system was introduced to dramatically promote the sensitivity of an immunoassay for ochratoxin A (OTA) detection. Specifically, a biotinylated M13 bacteriophage was introduced as a biofunctional competing antigen, in which a seven-peptide OTA mimotope fused on the p3 protein of M13 was used to specifically recognize an anti-OTA monoclonal antibody, and the biotin molecules modified on capsid p8 proteins were used in loading numerous streptavidin-labeled polymeric horseradish peroxidases (HRPs). Owing to the abundance of biotinylated p8 proteins in M13 and the high molar ratio between HRP and streptavidin in streptavidin-polyHRP, the loading amount of HRP enzymes on the M13 bacteriophage were greatly boosted. Hence, the proposed method exhibited high sensitivity, with a limit of detection of 2.0 pg/mL for OTA detection, which was 250-fold lower than that of conventional ELISA. In addition, the proposed method showed a slight cross-reaction of 2.3% to OTB, a negligible cross-reaction for other common mycotoxins, and an acceptable accuracy for OTA quantitative detection in real corn samples. The practicability of the method was further confirmed with a traditional HRP-based ELISA method. In conclusion, the biotinylated bacteriophage and polyHRP structure showed potential as a cascade-amplifying enzyme loading system for ultra-trace OTA detemination, and its application can be extended to the detection of other analytes by altering specific mimic peptide sequences.

## 1. Introduction

Ochratoxin A (OTA) is a secondary metabolite produced by *Penicillium* and *Aspergillus* [1] and shows high nephrotoxicity, hepatotoxicity, immunotoxicity, teratogenicity, carcinogenicity, and mutagenicity [2]. OTA contamination in a variety of crops, including corn, wheat, beans, coffee, and cocoa, is one of the major food safety issues that poses a serious health threat and causes substantial economic loss [3]. As such, to keep contaminated foods or feedstuffs off the food chain, many countries and international organizations have established strict limit standards for OTA residues [4]. For instance, the maximum residue levels of OTA in different food commodities ranges from 0.5 μg/kg to 10 μg/kg in the European Union standards. Various chromatography techniques, including high-performance liquid chromatography (HPLC) [5], liquid chromatography-mass spectroscopy [6], and gas chromatography-mass spectroscopy [7]), and immunoassays such as immunochromatographic assay [8] and enzyme-linked immunosorbent assay (ELISA) [9], have been adopted to guarantee the highly efficient monitoring of OTA. Chromatography techniques are sensitive and accurate but have limited use in rapid screening and detection due to some obvious drawbacks, including complex pretreatment process, high time requirement, and high cost. By contrast, immunologic methods are widely used as screening tools because of their excellent specificity, simplicity, rapidity, and low cost. ELISA is the most popular screening platform because it has a high throughput and excellent specificity, and is robust and easy to automate [10].

In conventional immunoassays for mycotoxins, such as OTA, a coating or the competing antigen of a hapten-protein/enzyme conjugate is usually chemo-synthesized through “trial and error”. This process may pose ecological and occupational threats due to the toxicity of hapten and the overconsumption of organic solvents [11]. Moreover, this process suffers from low conjugation efficiency because of extensive modification and blocking stages [12] and would inevitably lead to lot-to-lot variation [13]. Hence, several surrogates for competing antigens, such as anti-idiotypic antibodies [14], and mimotopes [15], have been proposed. Mimotopes are peptides that can mimic antibody-binding sites on antigens and are regarded as attractive antigen surrogates because they offer various possibilities for genetic engineering and low-cost production through in vitro bacterial expression [16]. Therefore, mimotopes are used as hapten conjugate substitutes and in establishing eco-friendly immunoassays for various mycotoxins [17,18,19]. However, similar to conventional ELISA, mimotope-based ELISA has low detection sensitivity due to the one-to-one stoichiometric ratio between horseradish peroxidase (HRP) and a target molecule in a competing binding event [19]. In theory, the high stoichiometric ratio between HRP and a competing antigen or antibody can enhance the colorimetric signal intensity. Thus, ensuring high enzyme loading on a competing antigen or antibody is a promising strategy for exponentially increasing the sensitivity of an immunoassay [20].

M13 bacteriophage (M13), a filamentous virus with 2700 identical copies of the major p8 protein and 3–5 copies of minor p3, p6, p7, and p9 proteins at both ends, is an attractive biological material that can be a solution for the above problems [21]. Through a gene modification-fusion expression process, mimotopes can be integrated on the N-terminals of p3 proteins; this process endows an M13 phage with mimicking ability for target antigen [22,23,24]. In addition, the detection sensitivity can be amplified by extensively functionalizing the abundant copies of major p8 proteins as the high-capacity containers of signal transducers or regulators (e.g., fluorescent dyes and enzymes) [25,26,27,28]. For example, Fang et al. [28] adopted a sulfhydryl-modified M13 phage as high-density container of Au@Ag nanozymes to enhance the sensitivity of the colorimetric biosensor.

In our previous report [29], an OTA mimicking M13 phage with an OTA mimotope fused on p3 proteins, termed as M13_OTA_, was panned from a seven-mer peptide phage display library by using an anti-OTA monoclonal antibody as a receptor. Herein, an eco-friendly ELISA for the ultratrace analysis of OTA in real corn was developed, wherein biotinylated M13_OTA_ phage was applied as a competing antigen and a container for cascade amplifying the loading of HRP enzymes. Some key parameters in the proposed cascade-amplifying enzyme loading based immunoassay (Bio-M13_OTA_-ELISA) were optimized. Analytical performance with respect of the 50% competitive inhibition concentration (IC_50_), limit of detection (LOD), accuracy, and reliability were evaluated and compared with those of the conventional HRP-based ELISA method.

## 2. Results and Discussion

### 2.1. Principle of the Proposed Bio-M13_OTA_-ELISA Method

**Scheme 1:**
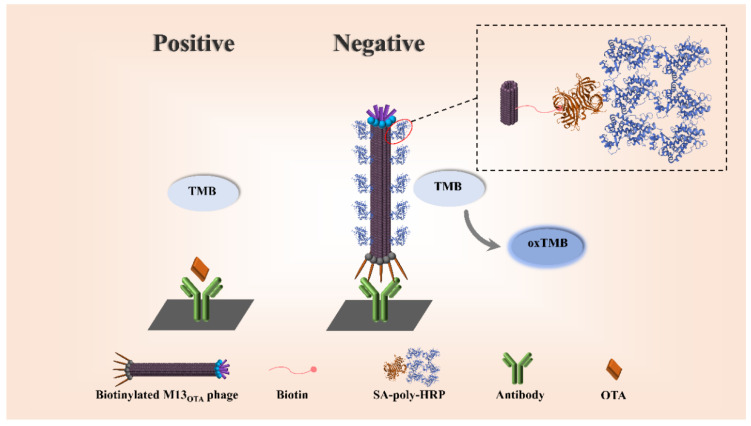
Biotin-M13_OTA_ as SA–polyHRP container for enhancing colorimetric immunoassay.

Herein, an ultrasensitive colorimetric immunoassay for OTA detection was established by integrating the functions of the M13 phage as a competing antigen and high-capacity container (Scheme 1). In detail, the M13_OTA_ phage was modified with numerous biotin molecules on the p8 proteins to serve as a high-capacity container for loading streptavidin-labeled HRP. To further increase the labeling amount of HRP on the competing antigen of the M13_OTA_ phage, a streptavidin–polymeric HRP (SA–polyHRP) [30] with an HRP-to-streptavidin ratio of 40 was used. Owing to the high loading amounts of HRP enzymes on the “phage–biotin–streptavidin–polymer” multilevel structure, the stoichiometric ratio between HRP and OTA in each competing event dramatically increased, and thus detection sensitivity greatly improved.

### 2.2. Preparation of Bio-M13_OTA_ Phage

**Figure 1:**
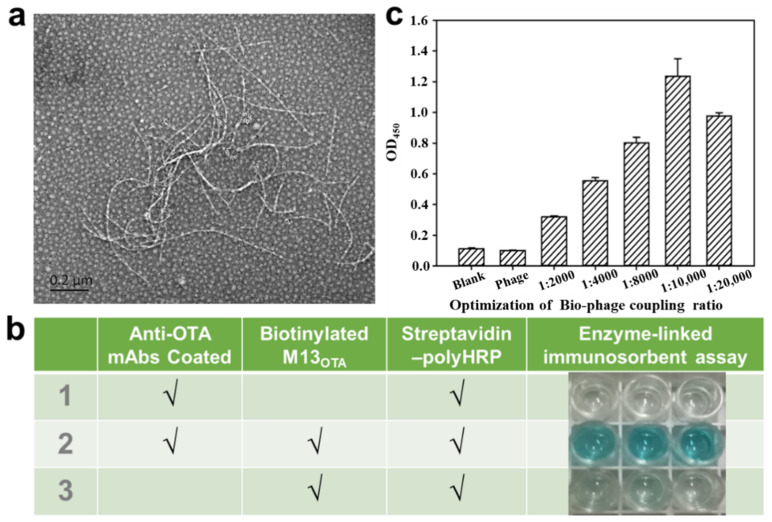
(**a**) TEM of M13_OTA_ bacteriophages; (**b**) proof of the concept of Bio-M13_OTA_ ELISA, “√” in the second column represents the plate wells coated with anti-OTA mAbs, the “√” in the second column represents the plate wells added with biotinylated M13_OTA_, and the “√” in the third column represents the plate wells added with streptavidin-polyHRP; (**c**) optimization of the coupling ratio of sulfosuccinimidyl biotin to M13_OTA_.

In our previous work, an M13_OTA_ bacteriophage was panned from a commercial phage display seven-peptide library, where the OTA mimotope peptide sequence of “GMSWMMA” was fused on the p3 proteins to endow the phage selectivity toward anti-OTA mAb [29]. The titer of the amplified M13_OTA_ was tested with a plate count method [28]. As shown in Figure S1, 10 μL of 10^8^-fold diluted phage solution developed 340 plaques of infected *E. coli* cells on the plates, indicating that the stock solution had a titer of 5.6 × 10^11^ pfu/mL. Thereafter, biotin molecules were modified on the p8 proteins of the M13_OTA_ phage (Figure 1a). Then, the designed proof of the concept assays was conducted to demonstrate the bifunction of the resultant Bio-M13_OTA_ as recognition agent and container of streptavidin-polyHRP. As shown in Figure 1b, a blue color developed on the anti-OTA mAb-coated microplates after incubation with Bio-M13_OTA_ and streptavidin-polyHRP. A colorless solution was obtained in the absence of Bio-M13_OTA_, indicating the binding abilities of Bio-M13_OTA_ to both anti-OTA mAbs and streptavidin. The colorless solution on the anti-OTA mAbs-free microplates indicated the neglectable biofouling of the phage and streptavidin. To achieve the optimal loading capacity of Bio-M13_OTA_, the effect of dosage ratio between M13_OTA_ and sulfosuccinimidyl biotin on the catalytic performance of Bio-M13_OTA_–streptavidin–polyHRP complex was assessed. Immunoassays were conducted for the development of “antibody–phage–polyHRP” structures on microplates coated with anti-OTA antibodies, and the resultant optical density at 450 nm (OD_450_) were detected. Figure 1c shows that the OD_450_ value increased when the dosage ratio between M13_OTA_ and sulfosuccinimidyl biotin changed from 1:2000 to 1:10,000, revealing that the loading capacity of Bio-M13_OTA_ increased with devoted biotin. However, the OD_450_ value decreased inversely when the amount of biotin modified on the phage increased, and this effect may have accounted for the steric effects of the overloaded polyHRP on the tetramethylbenzidine (TMB) substrates [31]. Hence, an optimal Bio-M13_OTA_ for carrying streptavidin–polyHRP was obtained through biotinylation at a M13_OTA_-to-sulfosuccinimidyl biotin dosage ratio of 1:10,000.

### 2.3. Development of Bio-M13_OTA_-ELISA

ELISA used Bio-M13_OTA_ as the competing antigen and a streptavidin–polyHRP container that can high-efficiently catalyze the oxidation of TMB to develop Bio-M13_OTA_ ELISA. First, the concentrations of anti-OTA ascites for the coating of 96-well microplates and Bio-M13_OTA_ were optimized with a checkerboard method. The immunoassays were performed at different conditions, and the resultant OD_450_ values of OTA-negative samples and OTA-positive samples (0.2 ng/mL) were recorded. As displayed in Table S1, the optimum concentrations of anti-OTA mAb and Bio-M13_OTA_ were 4 μg/mL and 1.1 × 10^9^ pfu/mL, respectively, and an acceptant OD_450_ value for OTA-negative sample and a relatively high inhibition rate of 0.2 ng/mL OTA were obtained under these conditions. The competing inhibition rate was calculated according to the formula of 1 − B/B_0_, where B and B_0_ represent the modified OD_450_ values (recorded value minus the background of the blank well) obtained from the detection of OTA-positive and OTA-negative samples, respectively.

**Figure 2:**
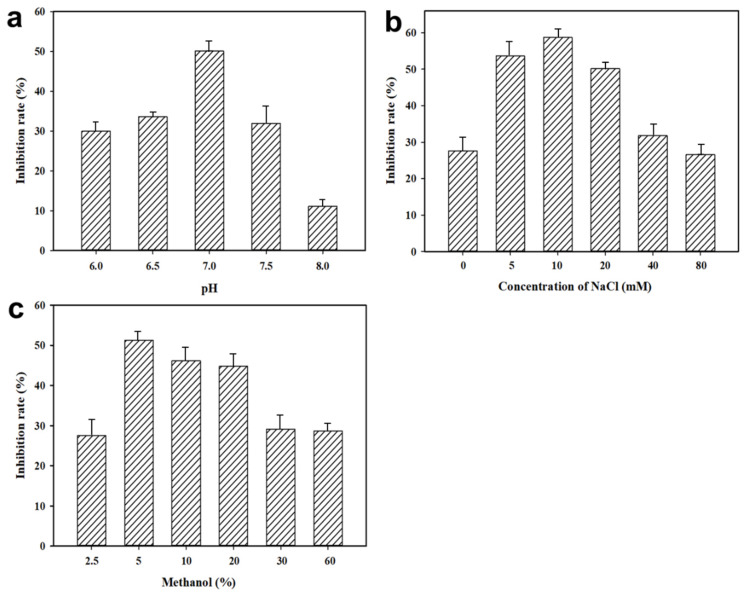
Optimization of the parameters of Bio-M13_OTA_ ELISA. (**a**) pH condition; (**b**) salt concentration (mM); (**c**) methanol content (%).

The pH, ionic strength, and methanol content of the sample solution could influence immuno-recognition interactions, which were then optimized based on the resultant competitive inhibition rates of 0.2 ng/mL OTA. The pH condition was optimized by adjusting the sample and Bio-M13_OTA_ solutions to a final pH value from 6.0 to 8.0, respectively; the ionic strength was optimized by adding NaCl solution in the sample and Bio-M13_OTA_ solutions to a final concentration from 0 mM to 80 mM, respectively, while the methanol concentration in the sample and Bio-M13_OTA_ solutions ranges from 2.5% to 60%. As shown in Figure 2a,b, the highest competitive inhibition rates of the proposed method were achieved when pH and NaCl concentration were 7.0 and 10 mM, respectively. An extract solution containing methanol was required for high extraction recovery from real samples because of the hydrophobic property of the OTA molecule. However, methanol can disrupt immune-recognition interactions. As revealed in Figure 2c, the competitive inhibition rate increased when the methanol content in a sample solution increased from 2.5% to 5%, whereas the competitive inhibition rate sharply decreased in the same sample at a higher methanol concentration. Hence, the appropriate buffer for preparing OTA standard sample was 10 mM Phosphate-buffered saline (PBS) buffer (pH = 7.0) containing 10 mM NaCl and 5% (*v*/*v*)methanol. Therefore, the extract solution of a real sample was diluted with 10 mM PBS buffer (pH = 7.0) until the final methanol content reached 5% (*v*/*v*).

### 2.4. Validation of Analytical Performance

**Figure 3:**
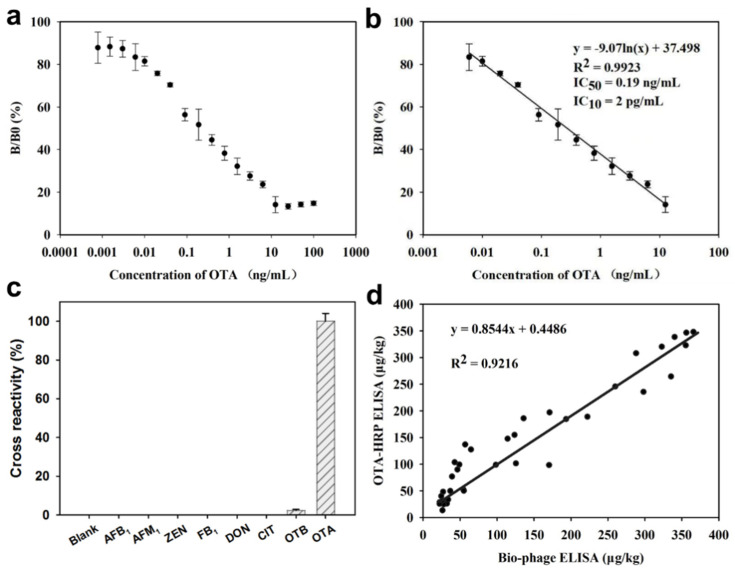
Validation of the analytical performance of Bio-M13_OTA_ ELISA. (**a**) The competitive inhibition curve of Bio-M13_OTA_ ELISA; (**b**) calibration curve of Bio-M13_OTA_ ELISA for OTA quantitative detection; (**c**) specificity of Bio-M13_OTA_ ELISA for OTA detection; (**d**) correlation between the results from Bio-M13_OTA_ ELISA and traditional ELISA; each test was replicated five times.

Under the above optimal conditions, the calibration curve of the developed Bio-M13_OTA_ ELISA was constructed by plotting the resultant OD_450_ values against the concentration of the OTA standard solution (0–100 ng/mL). As indicated in Figure 3a,b, the proposed method displayed good linearity and reliable correlation coefficient (R^2^ = 0.992) when the OTA concentration ranged from 0.006 ng/mL to 12.5 ng/mL. The linear regression equation was expressed as y = −0.089ln(x) + 0.36, where y is the OD_450_ value and x is the OTA concentration. The IC_50_ value was 0.19 ng/mL, and the LOD value was 2.0 pg/mL, which was equal to that of the 10% competing inhibition rate (IC_10_). These values were approximately 10-fold and 250-fold lower than those of conventional ELISA using OTA-HRP as competing antigen (IC_50_ = 1.8 ng/mL, IC_10_ = 0.5 ng/mL; Figure S3). Compared with streptavidin–HRP-based assay (Figure S4), the streptavidin–polyHRP with an HRP-to-streptavidin ratio of 40 increased the sensitivity with 9-fold and 75-fold decreased IC_50_ and LOD values, respectively. The results demonstrated the signal amplification effect of the container of Bio-M13_OTA_ and streptavidin–polyHRP.

The specificity of the newly type Bio-M13_OTA_ ELISA was evaluated by detecting the cross-reaction to the analogue of ochratoxin B (OTB), and other six common mycotoxins, including aflatoxin B_1_ (AFB_1_), aflatoxin M_1_ (AFM_1_), zearalenone (ZEN), fumonisin B_1_ (FB_1_), deoxynivalenol (DON), and citrinin (CIT). Figure 3c shows the proposed method exhibited the slight cross-reaction of 2.3% to OTB, and negligible cross-reaction to other common mycotoxins (less than 0.1%, Figure 3c). The precision and accuracy of the proposed method was assessed by analyzing OTA spiked corn samples at different concentrations ranging from 5 μg/kg to 50 μg/kg. The results in Table 1 show that the average recovery rates for the intra-assays ranged from 99.27% to 109.23% and coefficients of variation (CVs) of 4.10–9.91%. The recovery rates for the inter-assays varied from 97.83% to 101.46%, and the CVs were 5.09–13.18%. These results indicated that the accuracy and precision of the proposed method for OTA routine screening were acceptable. To further verify the reliability of the Bio-M13_OTA_ ELISA for OTA quantitative detection, a comparison study was performed between the conventional ELISA and proposed Bio-M13_OTA_ by blindly detecting 36 OTA fortified corn samples (Figure 3d). The results obtained using the two methods showed good agreement (R^2^ = 0.92), indicating that the proposed method is feasible for quantitative OTA detection in real corn samples.

## 3. Conclusions

A novel eco-friendly ELISA for detecting OTA was established by using Bio-M13 as the biological competing antigen. Owing to the cascade-amplifying HRP loading based on the high capacity of Bio-M13 and high ratio between HRP and streptavidin in streptavidin–polyHRP, the proposed method showed high sensitivity for OTA detection, with an LOD of 2.0 pg/mL, which was approximately 250-fold lower than that of the traditional ELISA method. Given that various target-related mimotopes, ligands, and nanobodies can be panned with the phage display technique, the proposed strategy has potential for the highly sensitive detection of small organic molecules, macromolecules, and even food-borne pathogens.

## 4. Materials and Methods

### 4.1. Materials

OTA, OTB, AFB_1_, AFM_1_, ZEN, FB_1_, DON, CIT, protein G, and sulfosuccinimidyl biotin were purchased from Sigma–Aldrich Chemical (St. Louis, MO, USA). SA–polyHRP, N-succinimidyl 3-(2-pyridyldithio) propionate, tri-(2-carboxyethyl)-phosphine hydrochloride, TMB, tween-20, and polyethylene glycol (PEG, Mw = 8000) were purchased from Wako Pure Chem., Inc. (Osaka, Japan). Luria–Bertani (LB) broth and Baird–Parker agar base were provided by Land Bridge Technology Co., Ltd. (Beijing, China). PBS solution (10×) was prepared by mixing 80 g of sodium chloride, 2 g of potassium chloride, 11.5 g of sodium hydrogen phosphate, and 2 g of potassium dihydrogen phosphate in 1 L of deionized water. Ultrapure water was produced using a Milli-Q system (Millipore, Milford, MA, USA). All the other chemical reagents (Sinopharm Chemical Corp., Shanghai, China) were of analytical grade and applied without further purification. The OTA-mimicking M13 bacteriophage (M13_OTA_) with a peptide sequence of “GMSWMMA” fused on its p3 proteins was prepared in our laboratory [29]. The 96-well microplates were obtained from Costar Inc. (Cambrige, MA, USA).

### 4.2. Propagation of M13_OTA_ Bacteriophage

*Escherichia coli* ER2738 cells were grown overnight in 5 mL of LB medium with a tetracycline concentration of 10 μg/mL at 37 °C. For the propagation of M13_OTA_ bacteriophages, 200 µL of LB medium containing *E. coli* cells and 1 µL of M13_OTA_ bacteriophage solution were inoculated in 20 mL of LB solution and cultured at 37 °C and 250 rpm for 4–5 h. After cell debris was removed through spinning at 5000 rpm for 10 min, M13_OTA_ bacteriophages in the supernatant were precipitated at 4 °C overnight by adding one-fifth of the volume of PEG/NaCl solution (2.5 M of NaCl and 20 wt.% PEG-8000). The precipitates were re-suspended in 1 mL of PBS, and cell debris was removed through spinning at 5000 rpm for 10 min. PEG/NaCl solution (200 µL) was mixed with the above supernatant and incubated on ice for 1.5 h. The M13_OTA_ bacteriophages were obtained through spinning at 12,000 rpm for 10 min. The pellets were re-suspended with 200 µL of PB buffer. The titer of the amplified M13_OTA_ bacteriophages was determined with the plate count method reported by Fang [28].

### 4.3. Preparation of Biotinylated M13_OTA_ (Bio-M13_OTA_) Bacteriophage

Bio-M13_OTA_ phages were prepared by coupling sulfosuccinimidyl biotin with the amine groups of capsid proteins, especially the major p8 protein, with the active ester method under an alkaline condition (PBS pH = 8.6). Briefly, sulfosuccinimidyl biotin was added to 1 mL of M13_OTA_ phage solution (5.6 × 10^11^ pfu/mL, pH = 8.6) and incubated on ice in the dark. Different dosage molar ratios between M13_OTA_ and sulfosuccinimidyl biotin (1:2000, 1:4000, 1:8000, 1:1:10,000, and 1:20,000) were evaluated, and the optimal loading capacity of Bio-M13_OTA_ for streptavidin-labeled enzyme was determined. After reacting for 4 h, the reaction fluid was dialyzed for 72 h with PB buffer and then stored at 4 °C.

### 4.4. Assay Procedure of Bio-M13_OTA_-ELISA for OTA Detection

The direct competitive ELISA for OTA detection was developed by using Bio-M13_OTA_ as the competing antigen. Briefly, 100 μL of protein G (25 μg/mL, pH 8.6) was added to the microplates and incubated at 4 °C overnight. After the microplates were washed three times with PBST (PBS solution containing 0.5% Tween-20), 100 μL of anti-OTA mAb solution (4 μg/mL, pH = 8.6) was added. The resulting solution was incubated at 37 °C for 1 h. After, excess anti-OTA mAb was removed by washing it three times with PBST, and the microplates were blocked with 5% skim milk (pH = 7.4) at 37 °C for 1 h. The microplates were then washed three times with PBST. Subsequently, 50 μL of the sample solution and 50 μL of Bio-M13_OTA_ (1.1 × 10^9^ pfu/mL) were added to each well, and the resulting solution was incubated for 1 h at 37 °C. After washing four times with with PBST, 100 μL of streptavidin–polyHRP (20 ng/mL in PBS, pH = 7.4) was added before another 1 h of incubation at 37 °C. After washing with unbonded streptavidin–polyHRP, 100 μL of substrate solution (10 mM TMB, 2 mM H_2_O_2_) was added, and the solution was incubated at 37 °C for 15 min. The chromogenic reaction was stopped by adding 50 μL of 2 M H_2_SO_4_, and the OD_450_ values were recorded on a microplate reader (Thermo Fisher, Vantaa, Finland).

### 4.5. Detection Performance of the Proposed Bio-M13_OTA_-ELISA for OTA Detection

The specificity of the Bio-M13_OTA_-ELISA was evaluated by detecting the cross-reaction to the analogue of OTB, and other six common mycotoxins, including AFB_1_, AFM_1_, ZEN, FB_1_, DON, and CIT, whereas the PBS solution containing 5 vt.% of methanol was used as a blank control. The cross-reaction rate is calculated according to the following formula: cross reaction rate (Cr%) = [(IC_50_ OTA)/(IC_50_ other mycotoxins)] × 100%. The accuracy and precision of the proposed Bio-M13_OTA_-ELISA were investigated by determining the average recoveries and variations of intra- and inter-assays. Five OTA spiked real corn samples, including low (5 and 10 ng/mL), medium (20 and 25 ng/mL), and high (50 ng/mL) levels of OTA concentrations, were prepared for intra- and inter-assays. Intra-assay was accomplished with 4 replicates at each concentration, while inter-assay was performed for 3 consecutive days with 4 replicates at each concentration. The reliability of the proposed method was assessed by determining the 36 OTA spiked real corn samples, and the results were further compared with those obtained from conventional HRP based-ELISA method. The OTA fortified corn samples were prepared by spraying the OTA solution with different concentrations into 36 real crushed corn samples. The weight of each OTA spiked sample was 5 g, and all samples were extracted for the tests using the proposed Bio-M13_OTA_-ELISA and conventional HRP based-ELISA, respectively.

### 4.6. Assay Procedure of Conventional HRP Based-ELISA for OTA Detection

Briefly, 100 μL of protein G (25 μg/mL, pH = 8.6) was added to the microplates and incubated at 4 °C overnight. After washing three times with PBST (PBS solution containing 0.5% Tween-20), 100 μL of anti-OTA mAb solution (1 μg/mL, pH = 8.6) was added, and incubated at 37 °C for 1 h. After removing the excess anti-OTA mAb by washing it three times with PBST, the microplates were blocked with 5% skim milk (pH 7.4) at 37 °C for 1 h, and then washed three times with PBST. Subsequently, 50 μL of the sample solution and 50 μL of OTA labeled HRP (0.5 μg/mL) were added to each well, and then incubated for 1 h at 37 °C. After washing three times with PBST, 100 μL of substrate solution (10 mM TMB, 2 mM H_2_O_2_) was added and incubated at 37 °C for 15 min. The chromogenic reaction was stopped by adding 50 μL of 2 M H_2_SO_4_, and the OD values at 450 nm were recorded on a microplate reader.

### 4.7. Corn Sample Pretreatment

Corn samples from grain procurement agencies in Shandong Province, China were validated to be OTA-free with the HPLC method. All the samples were ground and mixed well prior to the test. Sample extraction was performed according to our previous report. In Brief, 5 g of finely ground samples was mixed with 10 mL of PBS solution (pH = 7.4) containing 50% (*v*/*v*) methanol, and then the mixture was ultrasonically extracted for 20 min. After spinning at 10,000 rpm for 5 min, the supernatants were further diluted with PBS to a final methanol concentration of 5% before being detected.

## Figures and Tables

**Table 1:** Recovery and precision of Bio-M13_OTA_ ELISA in OTA-spiked corn samples.

OTA Added (μg/kg)	Intra-Assay Precision ^a^			Inter-Assay Precision ^b^		
	Mean (µg/kg)	Recovery (%)	RSD (%)	Mean (µg/kg)	Recovery (%)	RSD (%)
50	50.83 ± 0.04	101.66	5.85	50.73 ± 0.03	101.46	5.40
25	24.81 ± 0.03	99.27	4.10	24.45 ± 0.10	97.83	13.18
20	21.84 ± 0.07	109.23	9.86	20.15 ± 0.05	100.76	6.47
10	10.36 ± 0.05	103.60	5.67	10.14 ± 0.04	101.46	5.09
5	5.08 ± 0.09	101.67	9.91	5.04 ± 0.06	100.81	6.87

^a^ The assay was carried out in quadruplicates on the same day (*n* = 3). ^b^ The assay was performed on three consecutive days (*n* = 3).

## Data Availability

The data presented in this study are available on request from the corresponding author (X.C.), upon reasonable request.

## Supplementary Materials

The following are available online at https://www.mdpi.com/article/10.3390/toxins13110781/s1. Figure S1: M13 bacteriophage titer determination plate physical map; Table S1: Optimization of antibody and phage concentration by checkerboard titration method; Figure S2: The cross-reactivity of the proposed method; Figure S3: Calibration curve of conventional ELISA using OTA-HRP as competing antigen; Figure S4: Calibration curve of biotin-M13OTA ELISA using biotin-M13_OTA_-SA HRP as competing antigen.
