# Supplementary material for: Dramatically Enhancing the Sensitivity of Immunoassay for Ochratoxin A Detection by Cascade-Amplifying Enzyme Loading
Source: Toxins (Basel). 2021 Nov 5;13(11):781. doi: 10.3390/toxins13110781 (PMC8674760; doi:10.3390/toxins13110781)
Supplement: Supplementary file 1 [file toxins-13-00781-s001.zip › toxins-1434151 supplementary.pdf]

toxins

Supporting Information For:

Dramatically Enhancing the Sensitivity of Immunoassay for Ochratoxin A Detection by Cascade-Amplifying Enzyme Loading

Zhuolin Song [1,†], Lin Feng [1,†], Yuankui Leng [2,*], Mingzhu Huang [1], Hao Fang [2], Weipeng Tong [2], Xuelan Chen [1,*] and Yonghua Xiong [2]

[1] College of Life Science, Jiangxi Normal University, Nanchang 330022, China; zhuolinsong0508@163.com (Z.S.); linfeng2021102021@163.com (L.F.); huangmingzhu12@163.com (M.H.)
[2] State Key Laboratory of Food Science and Technology, Nanchang University, Nanchang 330047, China; 357900210001@email.nuc.edu.cn (H.F.); 40231337519017@email.ncu.edu.cn (W.T.); xiongyonghua@ncu.edu.cn (Y.X.)
* Correspondence: xuelanchen162@jxnu.edu.cn (X.C.); ykleng@ncu.edu.cn. (Y.L.)
† These two authors contributed equally to this work.

Citation: Song, Z.; Feng, L.; Leng, Y.; Huang, M.; Fang, H.; Tong, W.; Chen, X.; Xiong, Y. Dramatically Enhancing the Sensitivity of Immunoassay for Ochratoxin A Detection by Cascade-Amplifying Enzyme Loading. *Toxins* **2021**, *13*, 781. https://doi.org/10.3390/toxins13110781

Received: 8 October 2021
Accepted: 2 November 2021
Published: 5 November 2021

Publisher's Note: MDPI stays neutral with regard to jurisdictional claims in published maps and institutional affiliations.

Copyright: © 2021 by the authors. Submitted for possible open access publication under the terms and conditions of the Creative Commons Attribution (CC BY) license (http://creativecommons.org/licenses/by/4.0/).

1. Materials

1.1. LB Medium

Per liter: 10 g Bacto-Tryptone, 5 g yeast extract, 5 g NaCl.

1.2. LB IPTG/X-gal Stock

Mixing 1.25 g isopropyl-β-D-thioglactoside (IPTG) and 1 g 5-bromo-4-chloro-3-indoyl- β-D-glactoside (X-gal) in 25 mL dimethyl formamide (DMF). The obtained solution was stored at -20 °C for further use.

1.3. LB/ IPTG/X-gal Plates

Adding 15g agar to 1 liter of LB medium. After autoclaving, the LB medium was cooled to below 70°C, and 1 mL IPTG/Xgal was added and mixed well, then poured the plate and the plates were stored in the dark at 4°C for further use.

1.4. TOP Agar

Adding 7 g Bacto-Agar to 1 liter of LB Medium. After autoclaving, the medium was divided into 50 mL aliquots and stored at room temperature for further use.

2. M13 Titer Protocol

The number of plaques will increase linearly with added phage only when the multiplicity of infection (MOI) is much less than 1 (i.e., cells are in considerable excess). For this reason, it is recommended that phage stocks should be titered by diluting prior to infection, rather than by diluting cells infected at a high MOI. Plating at low MOI will also ensure that each plaque contains only one DNA sequence. Briefly, a swatch of *ER2738* cells grown on an LB/Tet plate was inoculated into 5–10 mL of LB and incubated with shaking 6–10 hours to mid-log phase (OD_{600} = 0.5). Then, 10 μL of the phages with 10 to 10^3-fold serial dilutions was used to infect the *ER2738* cells (200 μL). After incubation at room temperature for 5 minutes, the mixture was added into 3 mL of the top agar, and immediately poured culture onto a pre-warmed LB/IPTG/X-gal plate. The plates were cooled at room temperature for 5 minutes, inverted and incubated overnight at 37°C. Counting plaques (shown in Figure S1) on plates showed that 56 plaques from 10 μL of 10^8 dilution in the infected cells, indicating that the stock titer of phage solution was 5.6×10^{11} pfu/mL.

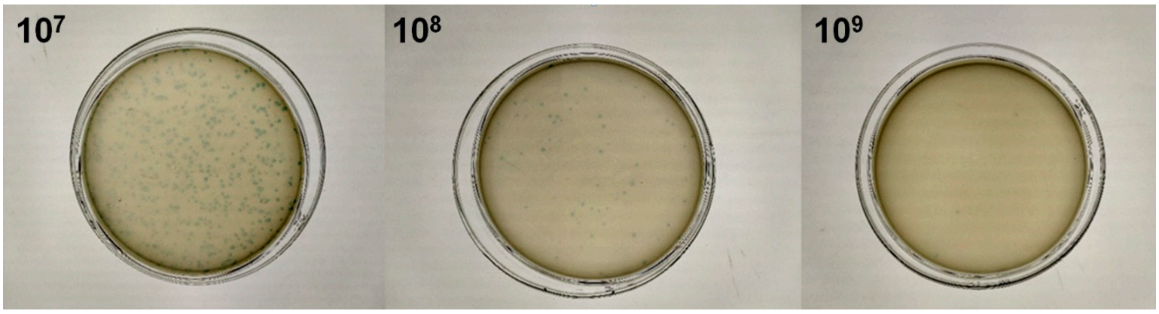

Figure S1. M13 bacteriophage titer determination plate physical map.

3. Optimization of antibody and phage concentration by checkerboard titration method

The concentrations of anti-OTA ascites for the coating of 96-well microplates and Bio-M13$_{OTA}$ were optimized with a checkerboard method. The immunoassays were performed at different conditions, and the resultant OD_{450} values of OTA-negative samples and OTA-positive samples were recorded. The results were displayed in Table S1.

Table S1 Optimization of antibody and phage concentration by checkerboard titration method (A) OTA free solution; (B) 0.2 ng/mL OTA standard solution; (C) the competitive inhibitory rate of 0.2 ng/mL OTA.

(A)

**Coated antibody concentration (μg/mL)**	**Concentration of Bio-M13$_{OTA}$ (pfu/mL)**					
	4.4×10^9	2.2×10^9	1.1×10^9	5.6×10^8	2.8×10^8	0
20	2.504	2.288	2.229	1.971	1.37	0.169
10	2.464	2.265	1.615	1.474	0.891	0.172
6.67	2.438	1.89	1.3	1.157	0.811	0.135
5	1.844	1.509	1.064	0.889	0.627	0.158
4	1.5	1.203	**1.055**	0.761	0.544	0.135
3.3	1.265	1.018	0.986	0.616	0.478	0.201
2.5	0.859	0.783	0.581	0.449	0.306	0.109
0	0.207	0.186	0.133	0.112	0.101	0.072

(B)

**Coated antibody concentration (μg/mL)**	**Concentration of Bio-M13$_{OTA}$ (pfu/mL)**					
	4.4×10^9	2.2×10^9	1.1×10^9	5.6×10^8	2.8×10^8	0
20	2.03	1.964	1.722	1.662	1.045	0.217
10	2.018	1.582	1.273	1.235	0.823	0.198
6.67	1.688	1.771	1.19	1.076	0.74	0.167
5	1.415	1.482	0.899	0.894	0.528	0.14
4	1.12	0.873	**0.652**	0.552	0.475	0.148
3.3	0.755	0.79	0.644	0.504	0.428	0.115
2.5	0.695	0.63	0.364	0.365	0.273	0.142
0	0.194	0.182	0.195	0.148	0.134	0.097

(C)

**Coated antibody concentration (μg/mL)**	**Concentration of Bio-$M13_{OTA}$ (pfu/mL)**				
	4.4×10^9	2.2×10^9	1.1×10^9	5.6×10^8	2.8×10^8
20	0.210	0.161	0.253	0.181	0.282
10	0.202	0.332	0.247	0.196	0.121
6.67	0.336	0.081	0.114	0.102	0.139
5	0.265	0.037	0.203	0.024	0.248
4	0.295	0.331	**0.463**	0.372	0.225
3.3	0.472	0.285	0.429	0.282	0.213
2.5	0.259	0.272	0.539	0.341	0.329

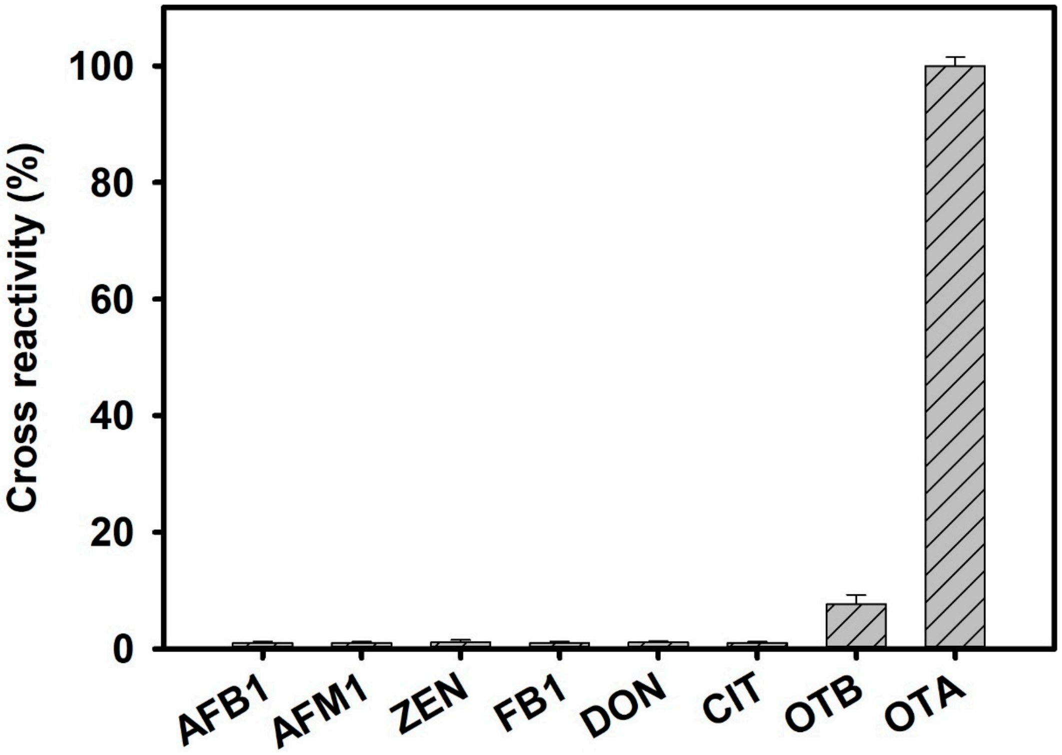

Figure S2. The cross-reactivity of OTA. The immunological cross reaction rate is calculated according to the following formula: cross reaction rate (Cr%) = [(IC_{50} DON)/(IC_{50} other mycotoxins)] × 100%.

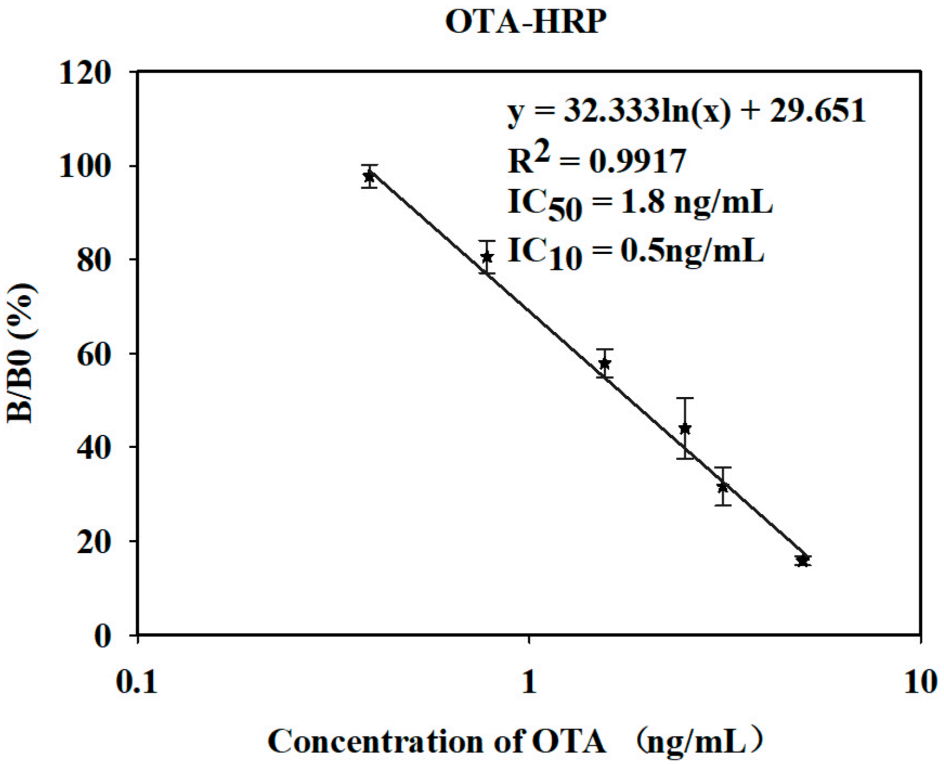

Figure S3. Calibration curve of conventional ELISA using OTA-HRP as competing antigen.
